# Supplementary material for: Cancer cell – Fibroblast crosstalk via HB-EGF, EGFR, and MAPK signaling promotes the expression of macrophage chemo-attractants in squamous cell carcinoma
Source: iScience. 2024 Aug 3;27(9):110635. doi: 10.1016/j.isci.2024.110635 (PMC11387794; doi:10.1016/j.isci.2024.110635)
Supplement: Document S1. Figures S1–S10 and Tables S1–S5 [file mmc1.pdf]

iScience, Volume *27*

Supplemental information

Cancer cell – Fibroblast crosstalk via HB-EGF, EGFR, and MAPK signaling promotes the expression of macrophage chemo-attractants in squamous cell carcinoma

Giovanni Giangreco, Antonio Rullan, Yutaka Naito, Dhruva Biswas, Yun-Hsin Liu, Steven Hooper, Pablo Nenclares, Shreerang Bhide, Maggie Chon U Cheang, Probir Chakravarty, Eishu Hirata, Charles Swanton, Alan Melcher, Kevin Harrington, and Erik Sahai

Supplementary figures

A

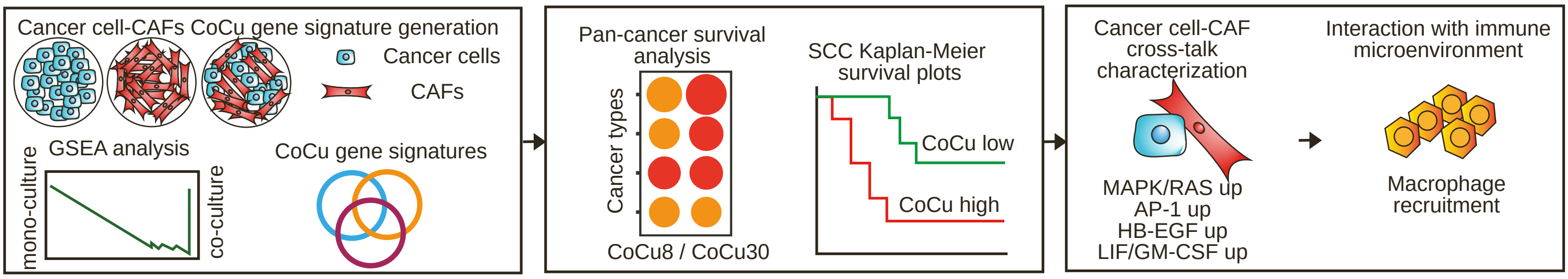

B

Transcriptomic data of cancer cells-CAFs mono-culture vs co-culture

↓

Selection of most upregulated genes for each mono-culture vs co-culture databases

↓

Selection of common genes upregulated in co-culture: CoCu30 gene signature

C

CoCu30 Gene Signature

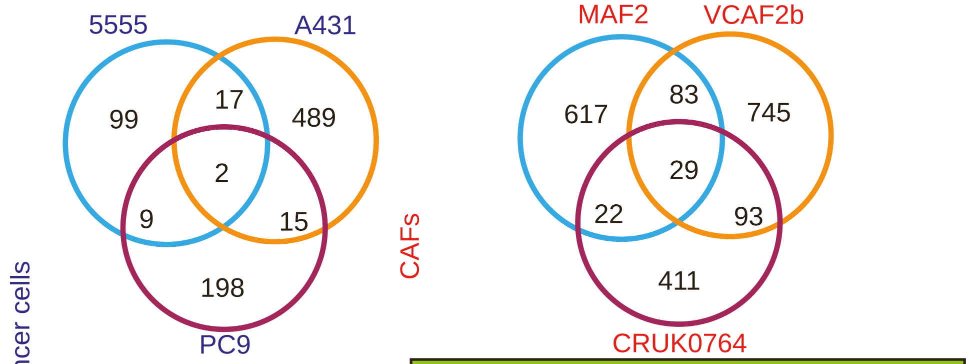

Gene
JUNB
FOSB

Gene			
ADAM19	FOSB	HHIP	MTHFD2
ASNS	GLIS3	IER3	MYO10
BAALC	GRB10	IFIT2	MTHFD2
CCL2	HBEGF	IL6	OAS2
F2RL1	HECW2	ITGA5	SERPINE1
FLNB	HES1	KCNN4	SPHK1
FOS	HES4	MLPH	TMEM51
			TRIB1

D

Cancer cell / CAF cell co-culture

NES	CoCu8	CoCu30
Wi38 with MDA-MB-231	-1.99	-2.30
CDD1112Sk with MDA-MB-231	-2.00	-2.12
HFF1 with MDA-MB-231	-2.05	-2.41
HFF2 with MDA-MB-231	-1.88	-2.15
MDA-MB-231 with Wi38	-1.91	-2.39
MDA-MB-231 with CDD1112Sk	-1.26	-1.48
MDA-MB-231 with HFF1	-1.89	-2.22
MDA-MB-231 with HFF2	-1.85	-2.07
Cal51 with Wi38	-1.92	-2.52
Cal51 with CDD1112Sk	-1.36	-1.76
Cal51 with HFF1	-1.97	-2.28
Cal51 with HFF2	-1.79	-1.82
Wi38 with Cal51	-1.33	-1.16
CDD1112Sk with Cal51	-1.56	-1.61
HFF1 with Cal51	-1.68	-1.97
HFF2 with Cal51	-1.69	-1.96

FDR<0.01
FDR<0.05
FDR n.s.

E

Cancer cell / endothelial cell co-culture

NES	CoCu8	CoCu30
HUVEC with 1205Lu	+0.73	-1.46
1205Lu with HUVEC	-1.12	-1.64

FDR<0.01
FDR<0.05
FDR n.s.

Figure S1

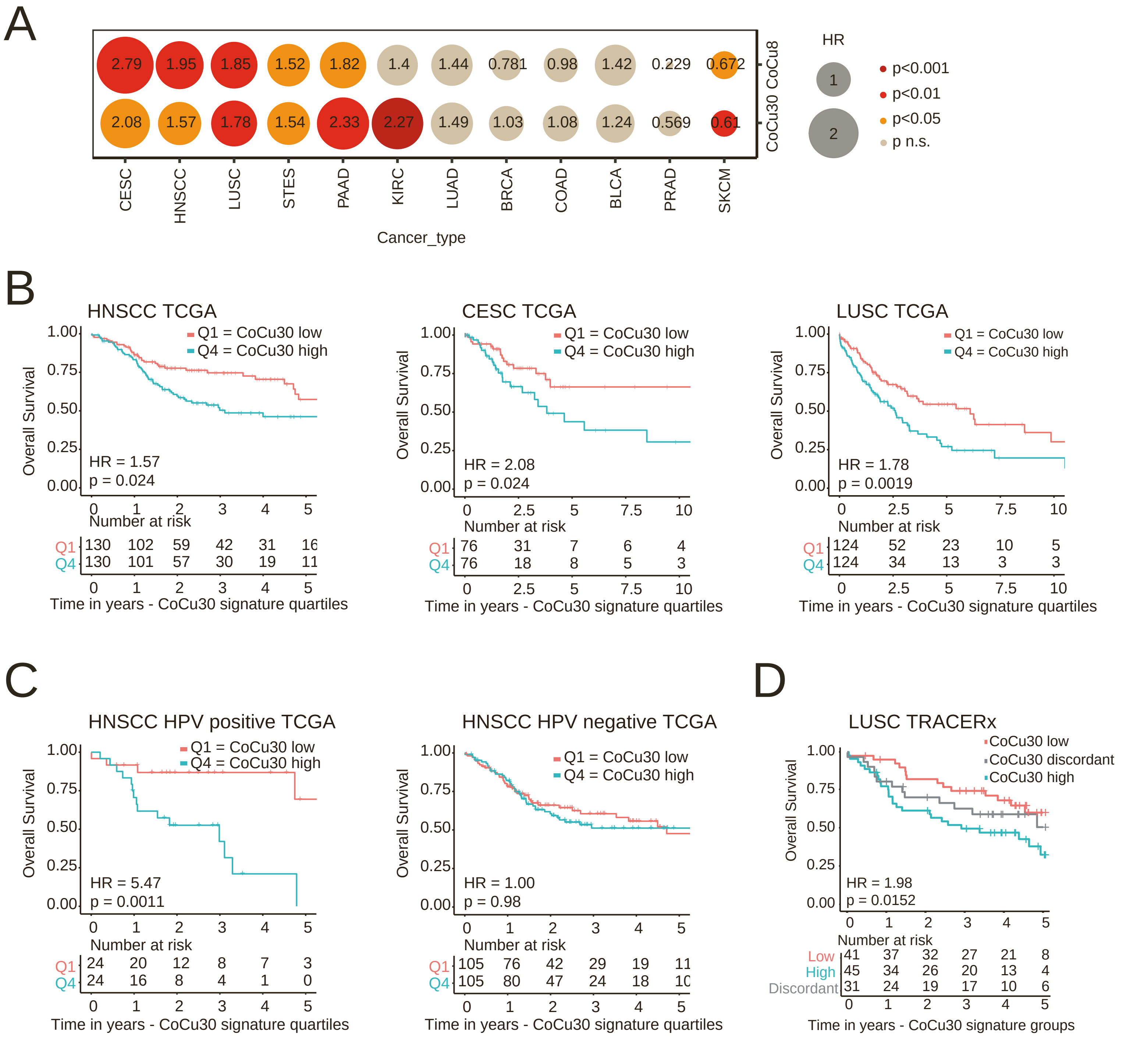
A
HR
p<0.001
p<0.01
p<0.05
p n.s.
CoCu30 CoCu8
CESC HNSCC LUSC STES PAAD KIRC LUAD BRCA COAD BLCA PRAD SKCM
Cancer_type
B
HNSCC TCGA
CESC TCGA
LUSC TCGA
Q1 = CoCu30 low
Q4 = CoCu30 high
Overall Survival
HR = 1.57
p = 0.024
HR = 2.08
p = 0.024
HR = 1.78
p = 0.0019
Number at risk
Time in years - CoCu30 signature quartiles
C
HNSCC HPV positive TCGA
HNSCC HPV negative TCGA
HR = 5.47
p = 0.0011
HR = 1.00
p = 0.98
D
LUSC TRACERx
CoCu30 low
CoCu30 discordant
CoCu30 high
HR = 1.98
p = 0.0152
Low
High
Discordant
Time in years - CoCu30 signature groups

Figure S2

CoCu8 Overall Survival

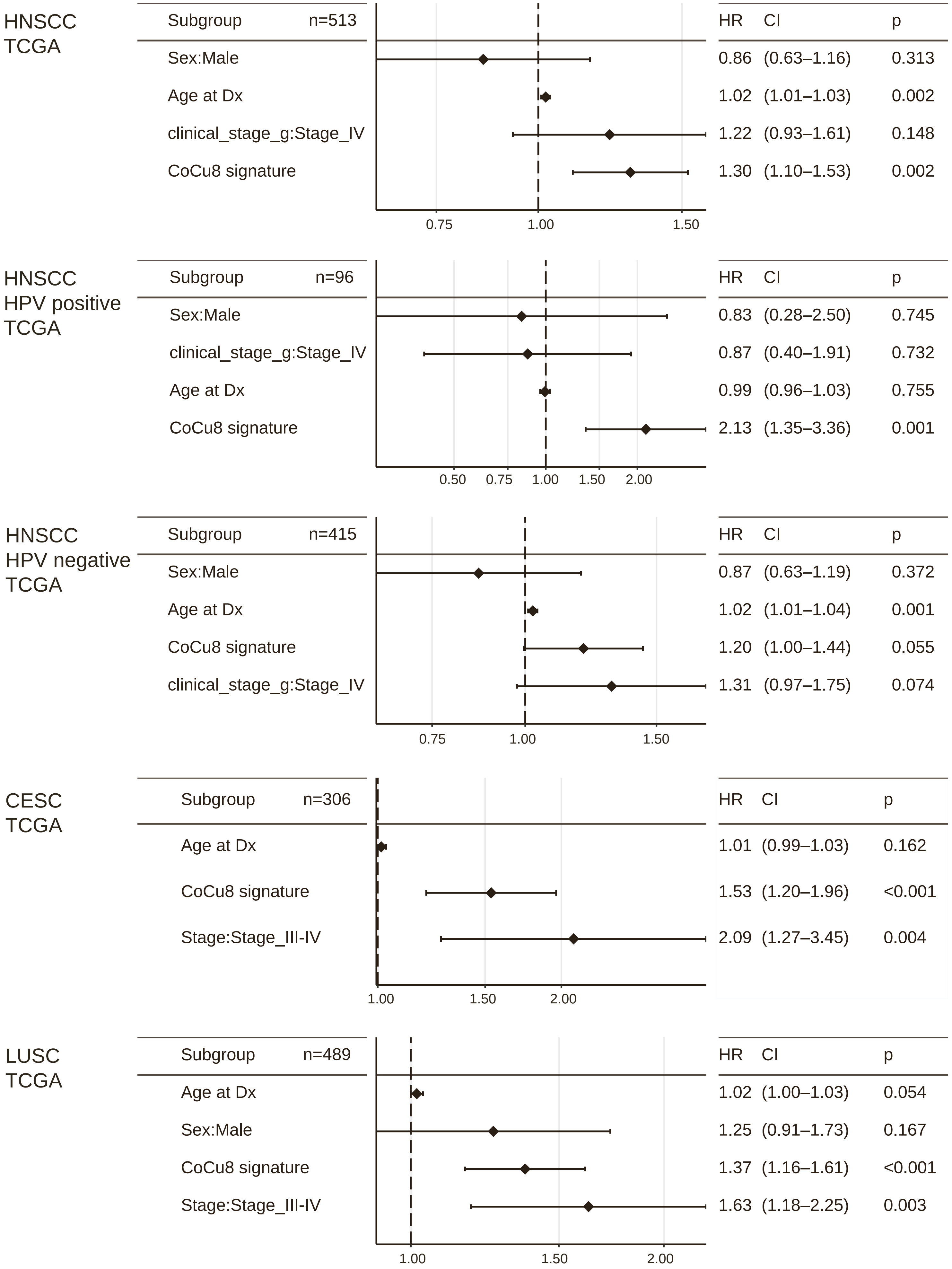

Figure S3

CoCu30 Overall Survival

HNSCC
TCGA

Subgroup (n=513)	HR	CI	p
Sex:Male	0.83	(0.61–1.12)	0.220
Age at Dx	1.02	(1.01–1.03)	0.002
clinical_stage_g:Stage_IV	1.23	(0.94–1.62)	0.130
CoCu30 signature	1.46	(1.09–1.95)	0.011

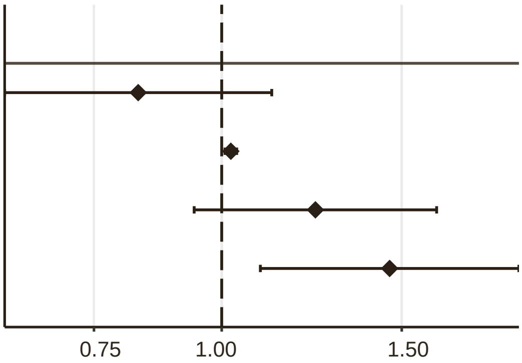

HNSCC
HPV positive
TCGA

Subgroup (n=96)	HR	CI	p
Sex:Male	0.81	(0.27–2.44)	0.713
clinical_stage_g:Stage_IV	0.95	(0.43–2.07)	0.892
Age at Dx	1.00	(0.96–1.03)	0.883
CoCu30 signature	4.48	(2.05–9.77)	<0.001

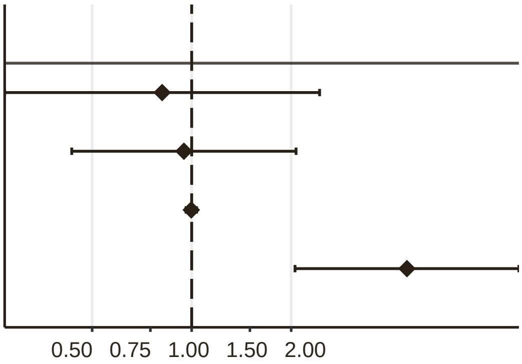

HNSCC
HPV negative
TCGA

Subgroup (n=415)	HR	CI	p
Sex:Male	0.85	(0.62–1.16)	0.306
Age at Dx	1.02	(1.01–1.04)	0.002
CoCu30 signature	1.19	(0.85–1.65)	0.312
clinical_stage_g:Stage_IV	1.32	(0.98–1.76)	0.066

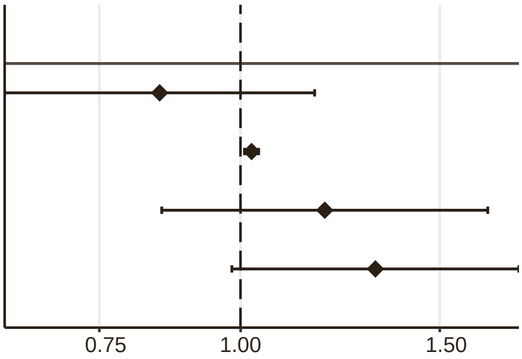

CESC
TCGA

Subgroup (n=306)	HR	CI	p
Age at Dx	1.01	(0.99–1.03)	0.239
CoCu30 signature	1.45	(0.92–2.30)	0.113
Stage:Stage_III-IV	2.20	(1.34–3.62)	0.002

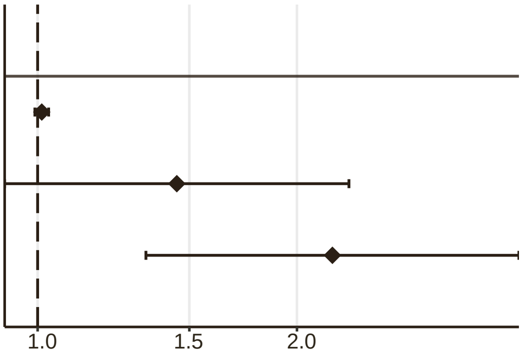

LUSC
TCGA

Subgroup (n=489)	HR	CI	p
Age at Dx	1.02	(1.00–1.03)	0.047
Sex:Male	1.30	(0.94–1.79)	0.111
Stage:Stage_III-IV	1.64	(1.19–2.26)	0.003
CoCu30 signature	1.72	(1.32–2.23)	<0.001

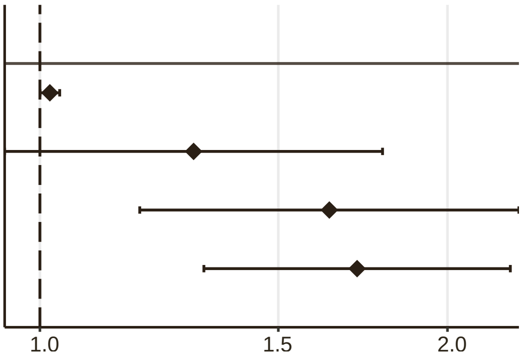

Figure S4

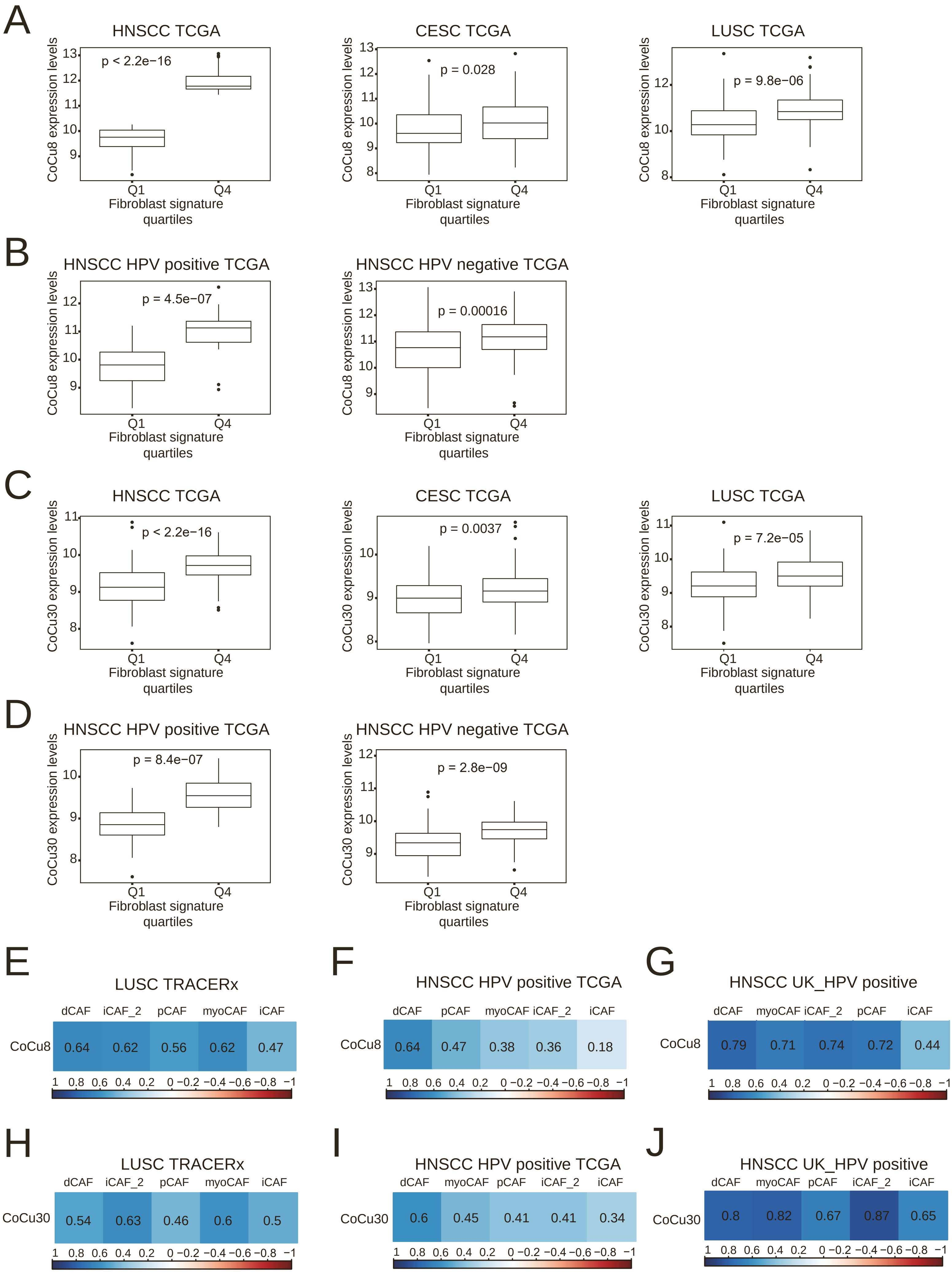
A
HNSCC TCGA
CoCu8 expression levels
p < 2.2e−16
CESC TCGA
p = 0.028
LUSC TCGA
p = 9.8e−06
Q1
Q4
Fibroblast signature quartiles
B
HNSCC HPV positive TCGA
p = 4.5e−07
HNSCC HPV negative TCGA
p = 0.00016
C
HNSCC TCGA
CoCu30 expression levels
p < 2.2e−16
CESC TCGA
p = 0.0037
LUSC TCGA
p = 7.2e−05
D
HNSCC HPV positive TCGA
p = 8.4e−07
HNSCC HPV negative TCGA
p = 2.8e−09
E
LUSC TRACERx
dCAF iCAF_2 pCAF myoCAF iCAF
CoCu8 0.64 0.62 0.56 0.62 0.47
F
HNSCC HPV positive TCGA
dCAF pCAF myoCAF iCAF_2 iCAF
CoCu8 0.64 0.47 0.38 0.36 0.18
G
HNSCC UK_HPV positive
dCAF myoCAF iCAF_2 pCAF iCAF
CoCu8 0.79 0.71 0.74 0.72 0.44
H
LUSC TRACERx
dCAF iCAF_2 pCAF myoCAF iCAF
CoCu30 0.54 0.63 0.46 0.6 0.5
I
HNSCC HPV positive TCGA
dCAF myoCAF pCAF iCAF_2 iCAF
CoCu30 0.6 0.45 0.41 0.41 0.34
J
HNSCC UK_HPV positive
dCAF myoCAF pCAF iCAF_2 iCAF
CoCu30 0.8 0.82 0.67 0.87 0.65
1 0.8 0.6 0.4 0.2 0 −0.2 −0.4 −0.6 −0.8 −1

Figure S5

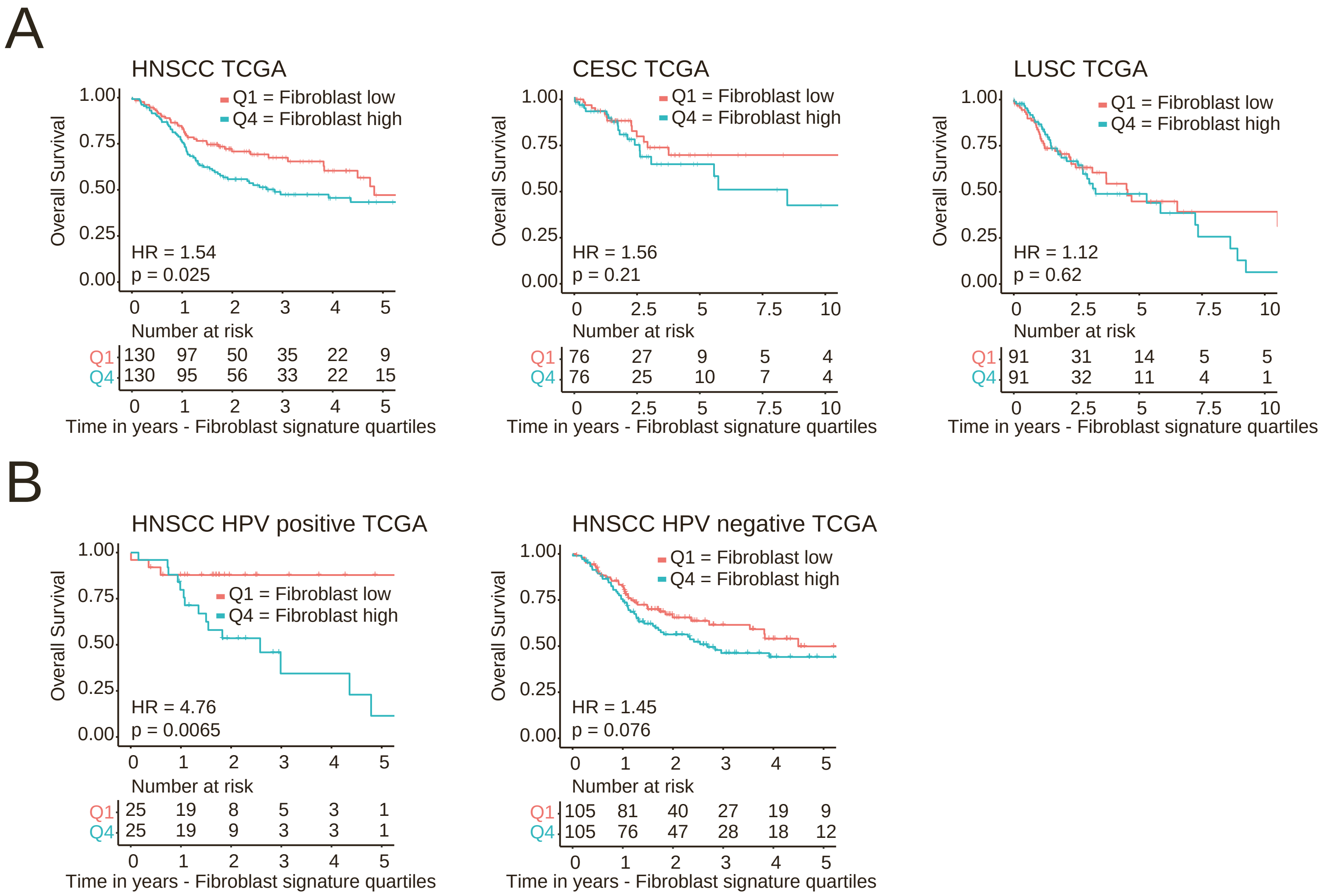
A
HNSCC TCGA
Q1 = Fibroblast low
Q4 = Fibroblast high
Overall Survival
HR = 1.54
p = 0.025
Number at risk
Q1 130 97 50 35 22 9
Q4 130 95 56 33 22 15
Time in years - Fibroblast signature quartiles
CESC TCGA
Q1 = Fibroblast low
Q4 = Fibroblast high
Overall Survival
HR = 1.56
p = 0.21
Number at risk
Q1 76 27 9 5 4
Q4 76 25 10 7 4
Time in years - Fibroblast signature quartiles
LUSC TCGA
Q1 = Fibroblast low
Q4 = Fibroblast high
Overall Survival
HR = 1.12
p = 0.62
Number at risk
Q1 91 31 14 5 5
Q4 91 32 11 4 1
Time in years - Fibroblast signature quartiles
B
HNSCC HPV positive TCGA
Q1 = Fibroblast low
Q4 = Fibroblast high
Overall Survival
HR = 4.76
p = 0.0065
Number at risk
Q1 25 19 8 5 3 1
Q4 25 19 9 3 3 1
Time in years - Fibroblast signature quartiles
HNSCC HPV negative TCGA
Q1 = Fibroblast low
Q4 = Fibroblast high
Overall Survival
HR = 1.45
p = 0.076
Number at risk
Q1 105 81 40 27 19 9
Q4 105 76 47 28 18 12
Time in years - Fibroblast signature quartiles

Figure S6

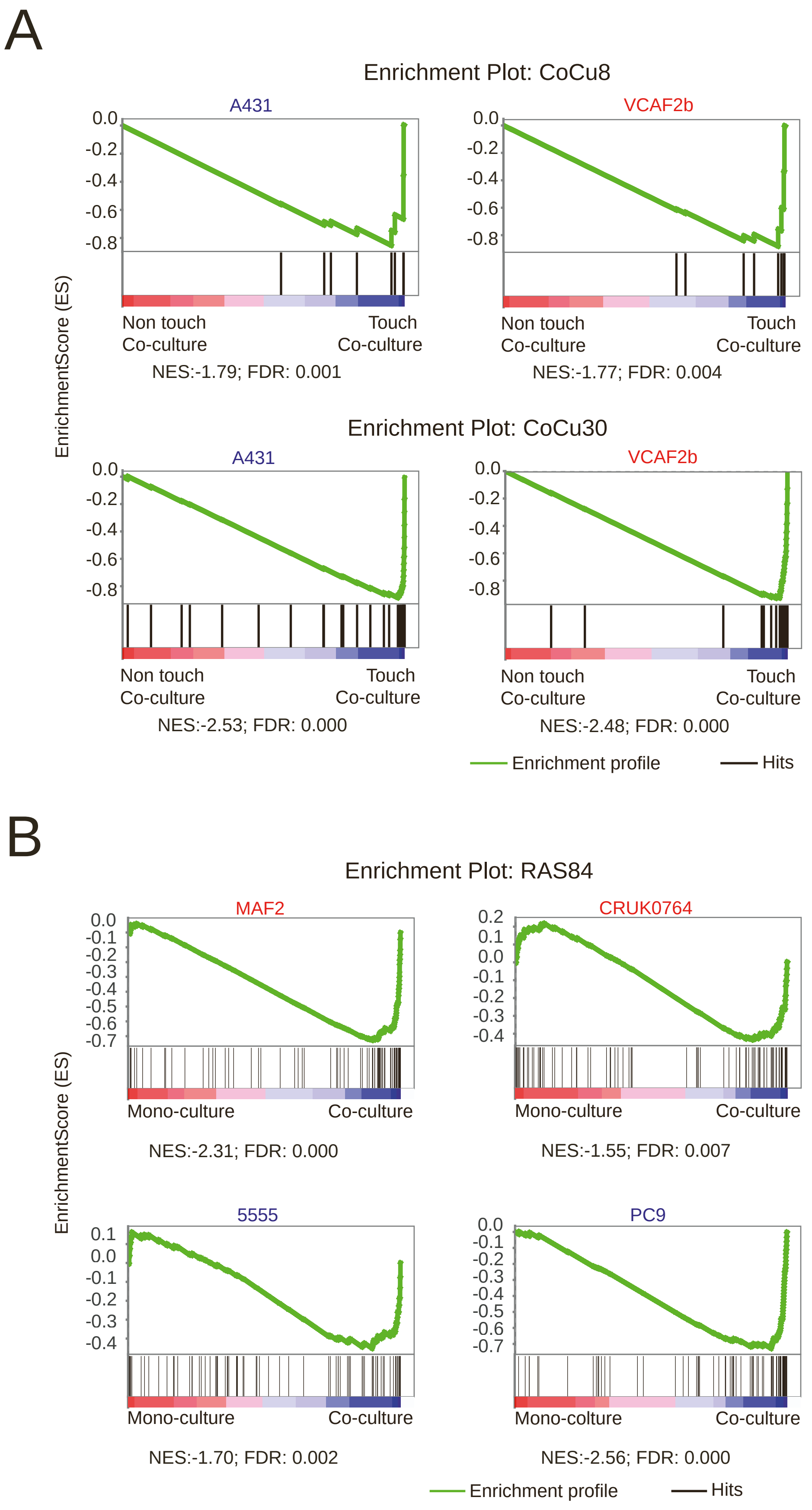
A
Enrichment Plot: CoCu8
A431
VCAF2b
0.0
-0.2
-0.4
-0.6
-0.8
Non touch Co-culture
Touch Co-culture
NES:-1.79; FDR: 0.001
NES:-1.77; FDR: 0.004
EnrichmentScore (ES)
Enrichment Plot: CoCu30
A431
VCAF2b
NES:-2.53; FDR: 0.000
NES:-2.48; FDR: 0.000
Enrichment profile
Hits
B
Enrichment Plot: RAS84
MAF2
CRUK0764
Mono-culture
Co-culture
NES:-2.31; FDR: 0.000
NES:-1.55; FDR: 0.007
5555
PC9
Mono-colture
NES:-1.70; FDR: 0.002
NES:-2.56; FDR: 0.000
EnrichmentScore (ES)
Enrichment profile
Hits

Figure S7

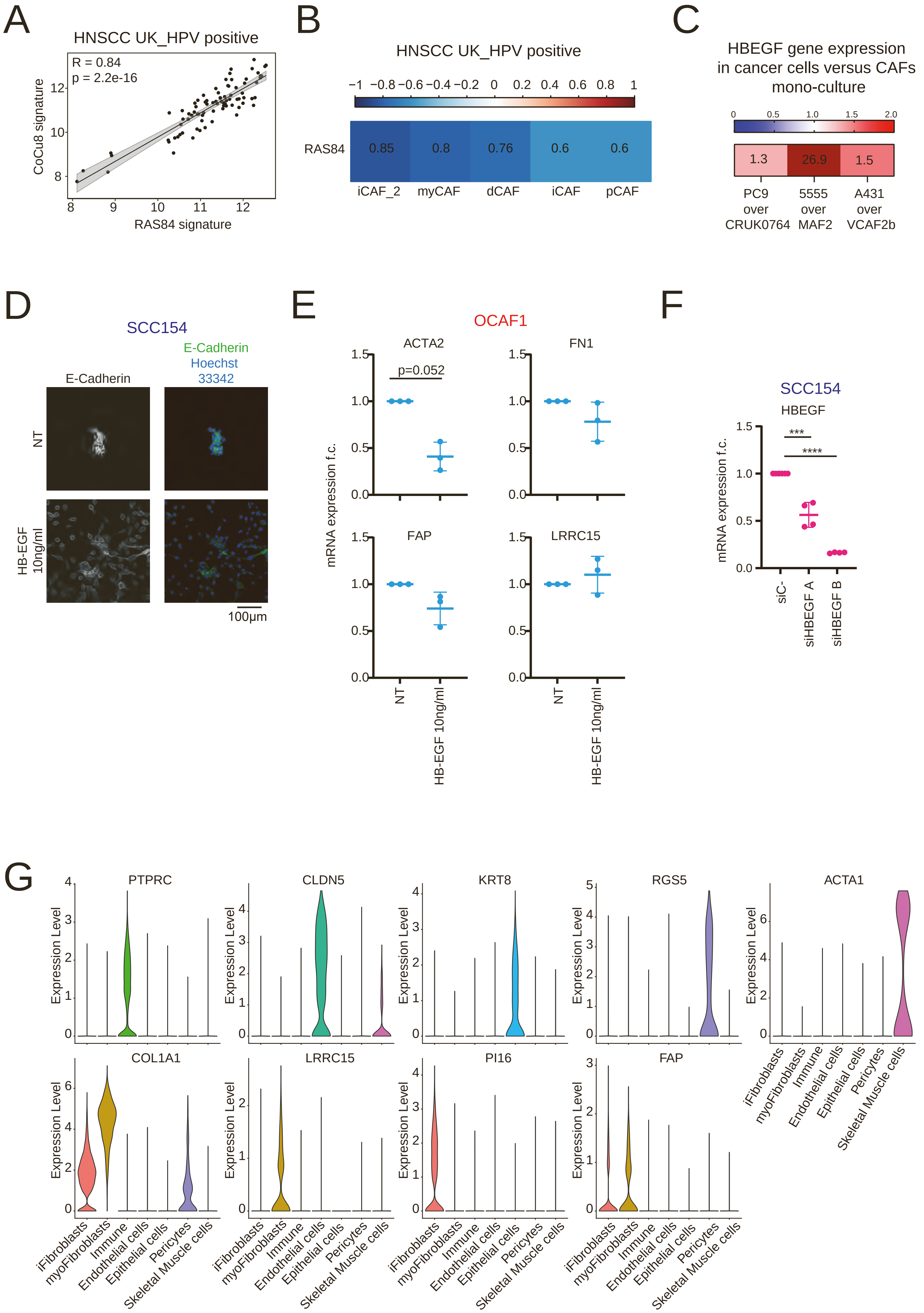
A
HNSCC UK_HPV positive
R = 0.84
p = 2.2e-16
CoCu8 signature
RAS84 signature
B
HNSCC UK_HPV positive
−1 −0.8 −0.6 −0.4 −0.2 0 0.2 0.4 0.6 0.8 1
RAS84
0.85 0.8 0.76 0.6 0.6
iCAF_2 myCAF dCAF iCAF pCAF
C
HBEGF gene expression in cancer cells versus CAFs mono-culture
0 0.5 1.0 1.5 2.0
1.3 26.9 1.5
PC9 over CRUK0764
5555 over MAF2
A431 over VCAF2b
D
SCC154
E-Cadherin
E-Cadherin Hoechst 33342
NT
HB-EGF 10ng/ml
100µm
E
OCAF1
ACTA2
p=0.052
FN1
FAP
LRRC15
mRNA expression f.c.
NT
HB-EGF 10ng/ml
F
SCC154
HBEGF
***
****
mRNA expression f.c.
siC-
siHBEGF A
siHBEGF B
G
PTPRC
CLDN5
KRT8
RGS5
ACTA1
COL1A1
LRRC15
PI16
FAP
Expression Level
iFibroblasts
myoFibroblasts
Immune
Endothelial cells
Epithelial cells
Pericytes
Skeletal Muscle cells

Figure S8

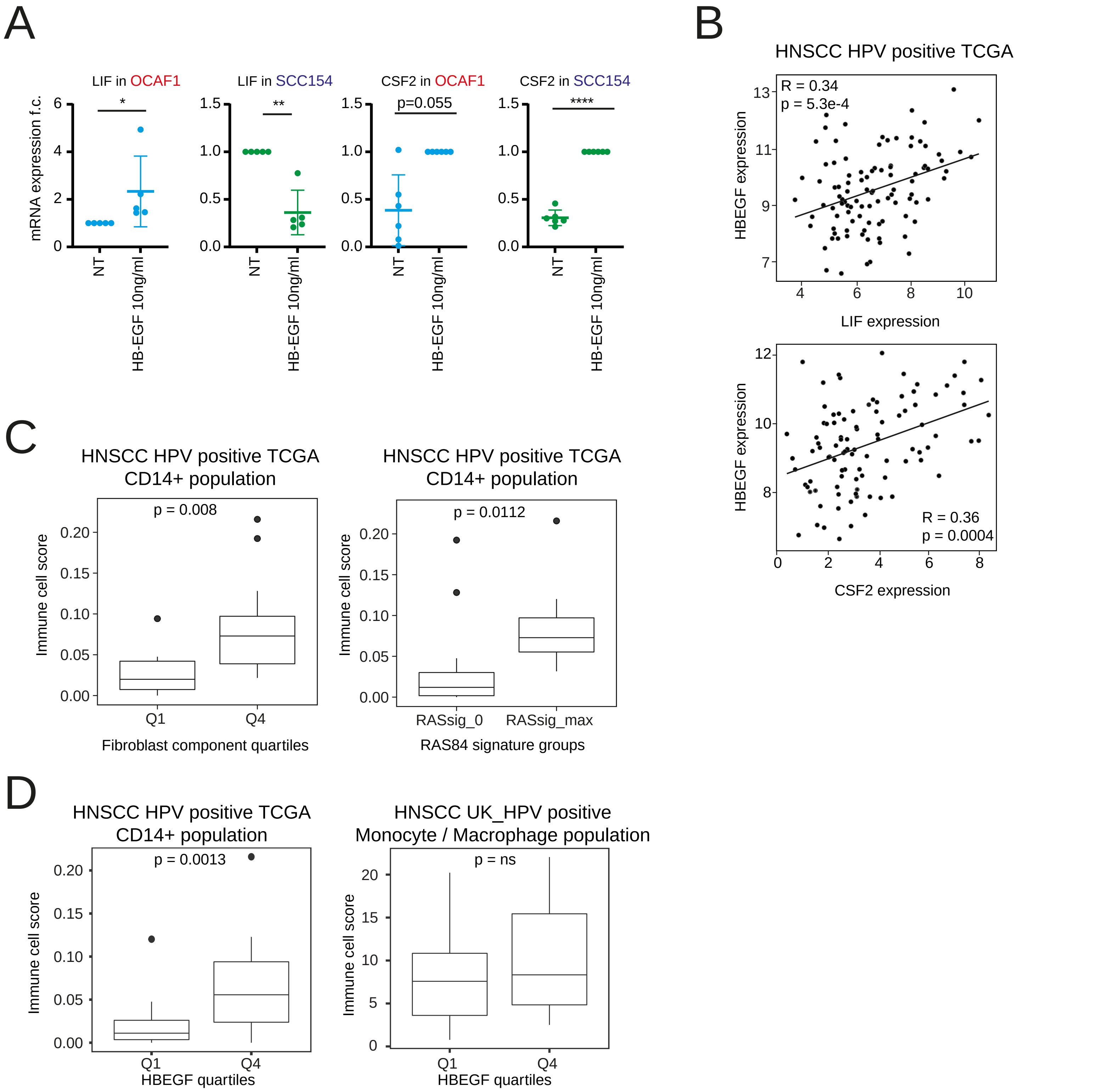

A
LIF in OCAF1
LIF in SCC154
CSF2 in OCAF1
CSF2 in SCC154
mRNA expression f.c.
*
**
p=0.055
****
NT
HB-EGF 10ng/ml
B
HNSCC HPV positive TCGA
R = 0.34
p = 5.3e-4
HBEGF expression
LIF expression
R = 0.36
p = 0.0004
CSF2 expression
C
HNSCC HPV positive TCGA
CD14+ population
p = 0.008
Immune cell score
Q1
Q4
Fibroblast component quartiles
HNSCC HPV positive TCGA
CD14+ population
p = 0.0112
Immune cell score
RASsig_0
RASsig_max
RAS84 signature groups
D
HNSCC HPV positive TCGA
CD14+ population
p = 0.0013
Immune cell score
Q1
Q4
HBEGF quartiles
HNSCC UK_HPV positive
Monocyte / Macrophage population
p = ns
Immune cell score
Q1
Q4
HBEGF quartiles

Figure S9

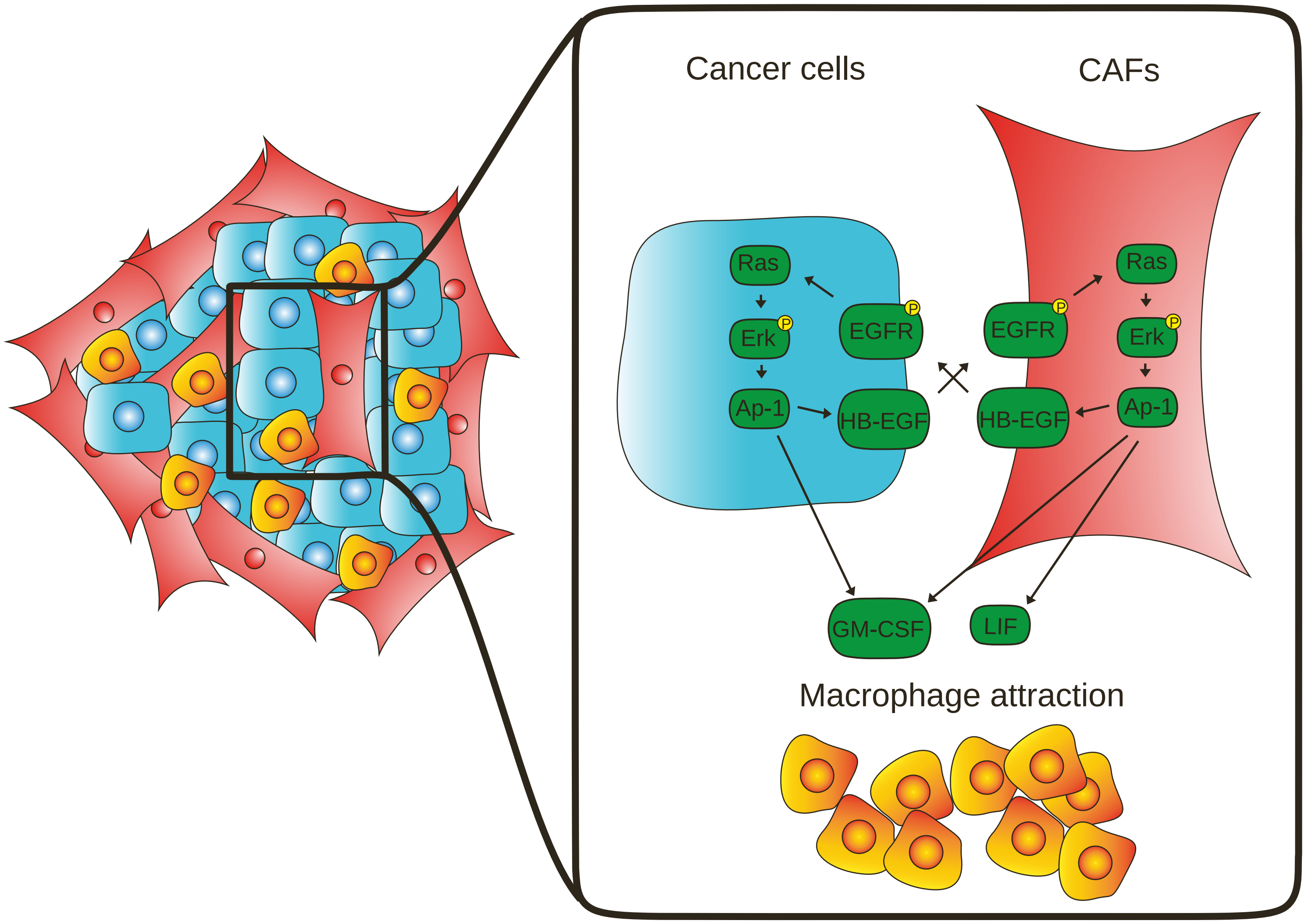
Cancer cells
CAFs
Ras
Erk
P
Ap-1
EGFR
P
HB-EGF
EGFR
P
HB-EGF
Ras
Erk
P
Ap-1
GM-CSF
LIF
Macrophage attraction

Figure S10

Supplementary figures title and legends

Figure S1: Generation and validation of CoCu8 / CoCu30 gene signatures, related to Figure 1. **A)** Flow chart description of the manuscript is provided. **B)** Strategy used to obtain CoCu30 gene signature. **C)** Venn diagram of the genes upregulated in the different datasets (top) and a table to summarize the genes constantly upregulated in the datasets (bottom) for cancer cells (left) and CAFs (right). **D)** Table with Normalized Enrichment Score (NES) values of different combinations of cancer cells and CAFs breast cancer cell lines from Rajaram et al. [21]. Negative values represent enrichment towards co-culture condition. Color legend is shown. **E)** Table with NES values of CoCu8 and CoCu30 gene signatures for cancer cells and endothelial cells mono-culture vs co-culture available from Stine et al. [22]. Negative values represent enrichment towards co-culture condition. FDR values and color legend are shown.

Figure S2: CoCu8 / CoCu30 gene signatures are associated with worse overall survival in multiple squamous cell carcinoma datasets, related to Figure 1. **A)** Bubble plot hazard ratios and p- values for overall survival for multiple TCGA cancer types using both CoCu8 and CoCu30 signatures. CESC - cervical squamous cell carcinoma; HNSCC - head and neck squamous cell carcinoma; LUSC – lung squamous cell carcinoma; PRAD - prostate adenocarcinoma; PAAD - pancreatic adenocarcinoma; LUAD - lung adenocarcinoma; KIRC - kidney clear cell carcinoma; COAD - colorectal adenocarcinoma; BLCA - bladder urothelial carcinoma; STES - esophagogastric carcinoma; SKCM – melanoma; BRCA - breast cancer. p-value ns is non-significant, * is p-value<0.05; ** is p-value<0.01, *** is p-value<0.001, **** is p-value<0.0001. **B)** Kaplan-Meier overall survival analysis of HNSCC (left), CESC (center), LUSC (right) TCGA datasets stratified for CoCu30 first vs last quartile. Below each analysis are shown the corresponding numbers at risk, time in years. HNSCC HR=1.57 (95%CI 1.06-2.35), p-value=0.024. CESC HR=2.08 (95%CI 1.09-4.01), p-value=0.024. LUSC HR=1.78 (95%CI 1.23-2.59), p-value=0.0019. HR and CI were calculated using Cox regression. p-value was calculated using logRank test. **C)** Kaplan-Meier overall survival analysis of HNSCC HPV positive (left) and HNSCC HPV positive (right) TCGA datasets stratified for CoCu30 first vs last quartile. Below each analysis are shown the corresponding numbers at risk, time in years. HPV positive HR=5.47 (95%CI 1.76-17.0), p-value=0.0011. HPV negative HR=1.00 (95%CI 0.67-1.51), p-value=0.98. HR and CI were calculated using Cox regression. p-value was calculated using logRank test. **D)** Kaplan-Meier overall survival analysis of LUSC TRACERx dataset. Individual tumors stratified as high-, discordant or low-risk according to expression profile of CoCu30 signature across multiple regions, as previously described and stratified according to Biswas et al. [53]. Below are shown the numbers at risk in years. HR=1.98 (95% CI 1.09-3.6), p-value=0.0152. HR and CI

calculated using Cox regression and are referred to CoCu30 low vs CoCu30 high. p-value was calculated using logRank test.

Figure S3: Multivariate analysis of CoCu8 overall survival, related to Figure 1. Forest plot showing Hazard Ratios, 95% confidence interval and p value calculated using multivariate Cox regression from patients with HNSCC (HPV positive and negative), LUSC and CESC from the TCGA cohort. Variables include: age (continuous, years), sex (male vs female, except for CESC as all patients were female), clinical stage (categorical) and the CoCu8 signature (continuous variable).

Figure S4: Multivariate analysis of CoCu30 overall survival, related to Figure 1. Forest plot showing Hazard Ratios, 95% confidence interval and p value calculated using multivariate Cox regression from patients with HNSCC (HPV positive and negative), LUSC and CESC from the TCGA cohort. Variables include: age (continuous, years), sex (male vs female, except for CESC as all patients were female), clinical stage (categorical) and the CoCu30 signature (continuous variable).

Figure S5: Fibroblast abundance correlates with CoCu8 / CoCu30 gene signature in different squamous cell carcinoma datasets, related to Figure 1. **A)** Box plot analysis of CoCu8 expression in HNSCC, CESC and LUSC TCGA separated by first and last quartile of fibroblast abundance via MethylCIBERSORT deconvolution strategy. Independent Student's t-test. (Left)p-value<2.2e-16, (middle) p-value-0.028, (right), p-value=9.8e-6. **B)** Box plot analysis of CoCu8 expression in HPV positive and negative TCGA separated by first and last quartile of fibroblast abundance via MethylCIBERSORT deconvolution strategy. Independent Student's t-test. (Left) p-value=4.5e-7, (right) p-value=0.00016. **C)** Box plot analysis of CoCu30 expression in HNSCC, CESC and LUSC TCGA separated by first and last quartile of fibroblast abundance via MethylCIBERSORT deconvolution strategy. Independent Student's t-test. (Left), p-value<2.2e-16, (middle) p-value=0.0037, (right) p-value=7.2e-5. **D)** Box plot analysis of CoCu30 expression in HPV positive and negative TCGA separated by first and last quartile of fibroblast abundance via MethylCIBERSORT deconvolution strategy. Independent Student's t-test. (Left) p-value=8.4e-7,(right) p-value=2.8e-9. **E-J)** Correlation plot of different fibroblast subpopulations derived from Galbo et al. [9] with CoCu8 gene signature in LUSC TRACERx **(E)**, HPV positive HNSCC TCGA **(F)**, UK_HPV positive HNSCC **(G)** patients and with CoCu30 gene signature in LUSC TRACERx **(H)**, HPV positive HNSCC TCGA **(I)**, UK_HPV positive HNSCC **(J)** patients. The number inside the square represents the R, Spearman correlation coefficient. The color legend is shown at the bottom. All correlations are significant p-value<0.05.

Figure S6: Fibroblast abundance meta-analysis on different squamous cell carcinoma datasets, related to Figure 1. **A)** Kaplan-Meier overall survival analysis of HNSCC (left), CESC (center), LUSC (right) TCGA datasets stratified for fibroblast abundance first vs last quartile. Below each analysis are shown the corresponding numbers at risk, time in years. HNSCC HR=1.54 (95%CI 1.05-2.25), p-value=0.025. CESC HR=1.56 (95%CI 0.77-3.17), p-value=0.21. LUSC HR=1.12 (95%CI 0.71-1.77), p-value=0.62. HR and CI were calculated using Cox regression. p-value was calculated using logRank test. **B)** Kaplan-Meier overall survival analysis of HNSCC HPV positive (left) and HNSCC HPV positive (right) TCGA datasets stratified for fibroblast abundance first vs last quartile. Below each analysis are shown the corresponding numbers at risk, time in years. HPV positive HR=4.76 (95%CI 1.36-16.5), p-value=0.0065. HPV negative HR=1.45 (95%CI 0.96-2.18), p-value=0.076. HR and CI were calculated using Cox regression. p-value was calculated using logRank test.

Figure S7: Enrichment of CoCu8, CoCu30 and RAS84 gene signatures in different co-culture conditions, related to Figures 2, 3. **A)** Gene set enrichment analysis (GSEA) plot of CoCu8 gene signature (top) and CoCu30 (bottom) in co-culture indirect vs direct condition in A431 / VCAF2b transcriptomic dataset. NES and FDR are specified below each plot. **B)** Gene set enrichment analysis (GSEA) plot of RAS84 gene signature [28] in mono-culture and co-culture. NES and FDR are specified below each plot.

Figure S8: HB-EGF/RAS/MAPK activity in cancer cells and CAFs mono-culture vs co-culture, related to Figures 3, 4, 5. **A)** Correlation plot of CoCu8 and RAS84 expression levels in HNSCC HPV positive UK_HPV positive cohort. R is Spearman correlation coefficient. P-value=2.2e-16. **B)** Correlation table of different fibroblast subpopulations derived from Galbo et al. [9] with RAS84 gene signature in the independent cohort UK_HPV positive of HNSCC HPV positive patients. The number inside the square represents the Spearman R, correlation coefficient. The color legend is shown on the top. All correlations are significant at p-value<0.001. n=97. **C)** Heatmap of expression of the *HBEGF* in all the tested transcriptomic datasets. Every box represents the fold change difference of each gene for the corresponding cancer cell line in mono-culture when compared to its corresponding CAF cell line in mono-culture. **D)** Immunofluorescence staining of E-Cadherin and Hoechst 33342 for SCC154 mono-culture for the indicated treatments after 48h. **E)** qPCR analysis of *ACTA2*, *FAP*, *FN1* and *LRRC15* genes in OCAF1 mono-culture for the indicated treatments after 48h. mRNA expression is reported as mean ± standard deviation (SD) fold change difference over non-treated (NT) condition. Genes have been normalized over the average of *GAPDH*, *ACTB* and *RPLP0* housekeeping genes. n = 3 independent experiments. Two tailed paired Student's t-test. p-value is indicated. **F)** qPCR analysis of *HBEGF* gene in SCC154 after 96h of treatment with the indicated conditions. mRNA expression is reported as

mean ± standard deviation (SD) fold change difference over siC- condition. Gene has been normalized over the average of *GAPDH*, *ACTB* and *RPLP0* housekeeping genes. n ≥ 4 independent experiments. Two tailed paired mixed effects model corrected for Holm-Sidak multiple comparison test. p-value *** is p-value<0.001. **F)** Violin plot of mRNA expression levels of the indicated genes for each cluster from Choi et al. [33] .

Figure S9: HB-EGF effect on cytokine production and monocyte/macrophage enrichment related to Figure 5. **A)** qPCR analysis of *LIF* and *CSF2* genes in OCAF1 and SCC154 mono-cultures for the indicated treatments after 48h. mRNA expression is reported as mean ± standard deviation (SD) fold change difference over non-treated (NT) condition for each cell type with *LIF*, while for *CSF2* fold change difference is reported over Hb-EGF 10ng/ml treated sample. Genes have been normalized over the average of *GAPDH*, *ACTB* and *RPLP0* housekeeping genes. n ≥ 5 independent experiments. Two tailed paired Student's t-test. p-value * is p-value<0.05, ** is p-value<0.01, **** is p-value<0.0001. **B)** Correlation plot of *HBEGF* with *LIF* (left) and *HBEGF* with *CSF2* (right) expression levels in HNSCC HPV positive TCGA dataset. R is Spearman correlation coefficient. (Left) p-value=5.3e-4, (right) p-value=0.0004. **C)** (Right) Box plot analysis of immune cell absolute score via MethylCIBERSORT deconvolution strategy in HNSCC HPV positive separated by first and last quartile of fibroblast abundance. Independent Student's t-test. p-value=0.008. (Left) Box plot analysis of immune cell absolute score via MethylCIBERSORT deconvolution strategy in HNSCC HPV positive separated by RAS84_0 and RAS84_max. Independent Student's t-test. p-value=0.0112. **D)** (Right) Box plot analysis of immune cell absolute score via MethylCIBERSORT deconvolution strategy in HNSCC HPV positive separated by first and last quartile of HBEGF expression. Independent Student's t-test. p-value=0/0013. (Left) Box plot analysis of immune cell absolute score via MethylCIBERSORT deconvolution strategy in HNSCC HPV positive separated by HBEGF expression. Independent Student's t-test. p-value ns is non-significant.

Figure S10: Schematic representation of the current cross-talk model of cancer cells – CAFs co-culture related to Figures 1, 2, 3, 4, 5.

Supplementary tables

Supplementary table 1

		TCGA HNSCC	TCGA LUSC	TCGA CESC	TCGA BLCA	TCGA BRCA	TCGA COAD	TCGA KIRC	TCGA LUAD	TCGA PAAD	TCGA PRAD	TCGA SKCM	TCGA STES	TracerX LUSC
Number of patients		513	495	306	413	1108	633	538	524	186	501	479	559	117
Age (median, years)		61	68	46	68	58	65	61	66	65	61	58	66	70
Sex	Males	379	366	0	305	12	297	347	244	103	501	296	405	81
	Females	134	129	306	108	1096	336	191	280	83	0	183	154	36
Race	Asian	11	9	20	44	61	12	8	8	11	2	12	0	0
	Black or African Ametican	47	29	31	23	186	67	57	54	7	7	1	0	0
	White	439	348	210	328	764	298	466	394	163	163	454	0	113
	Other / NA	16	109	45	18	100	256	7	68	5	5	13	559	4

Supplementary table 2

Markers	Cell line: OCAF1	Cell line: OCAF2	Cell line: 796T
FGA	19, 20	22, 26	19,24
TPOX	8, 11	8, 10	11,11
D8S1179	13, 14	14, 15	11,12
vWA	14, 17	15, 17	17,17
AMEL	X, Y	X, X	X,Y
Penta D	13, 14	9, 13	10,12
CSF1PO	11, 12	11, 12	10,11
D16S539	11, 13	11, 12	12,12
D7S820	11, 12	10, 11	8,10
D13S317	9, 13	11, 12	8,9
D5S818	11, 11	12, 12	12,12
Penta E	12, 12	11, 18	8,13
D18S51	14, 16	12, 16	11,15
D21S11	28, 31	27, 28	29,31
TH01	9.3, 9.3	6, 6	6,9.3
D2S1358	15, 19	15, 15	15,15

Supplementary table 3

GENE	Species	Entrez Gene ID	Primer direction	Sequence (5' to 3')
JUNB	Homo sapiens	3726	Forward	AACAGCCCTTCTACCACGAC
JUNB	Homo sapiens	3726	Reverse	CAGGCTCGGTTTCAGGAGTT
FOS	Homo sapiens	2353	Forward	TGACTGATACACTCCAAGCGG
FOS	Homo sapiens	2353	Reverse	GGCAATCTCGGTCTGCAAAG
FOSB	Homo sapiens	2354	Forward	CCGTTGAATTGGAAACTGCT
FOSB	Homo sapiens	2354	Reverse	CAGAGGGAGAGAGACCATCG
CXCL2	Homo sapiens	2920	Forward	CACAGTGTGTGGTCAACATTTC
CXCL2	Homo sapiens	2920	Reverse	CACAGAGGGAAACACTGCAT
CXCL8	Homo sapiens	3576	Forward	TCTGGCAACCCTAGTCTGCT
CXCL8	Homo sapiens	3576	Reverse	AAACCAAGGCACAGTGGAAC
LIF	Homo sapiens	3976	Forward	CCTTTCCCTGGTCCCTACTC
LIF	Homo sapiens	3976	Reverse	CACCCGTAAGGCTTATTCCA
CSF2	Homo sapiens	1437	Forward	AAATGTTTGACCTCCAGGAGCC
CSF2	Homo sapiens	1437	Reverse	ATCTGGGTTGCACAGGAAGTT
ACTB	Homo sapiens	60	Forward	AGAAAATCTGGCACCACACC
ACTB	Homo sapiens	60	Reverse	CAGAGGCGTACAGGGATAGC
GAPDH	Homo sapiens	2597	Forward	CAAAAGGGTCATCATCTCTGC
GAPDH	Homo sapiens	2597	Reverse	AGTTGTCATGGATGACCTTGG

RPLP0	Homo sapiens	6175	Forward	AACCCAGCTCTGGAGAAACT
RPLP0	Homo sapiens	6175	Reverse	CCCCTGGAGATTTTAGTGGT
ACTA2	Homo sapiens	59	Forward	ACCCTGTTCCAGCCATCCTT
ACTA2	Homo sapiens	59	Reverse	TGCCCCCTGATAGGACATTG
COL7A1	Homo sapiens	1294	Forward	GGGCATCCAGCTACATCCTA
COL7A1	Homo sapiens	1294	Reverse	GCTTGAGATCCCTGGAAGTG
FN1	Homo sapiens	2335	Forward	CAAAGCAAGCCCGGTTGTTA
FN1	Homo sapiens	2335	Reverse	CCCACTCGGTAAGTGTTCCC
LRRC15	Homo sapiens	131578	Forward	AGGGACGGACACTGTACCTG
LRRC15	Homo sapiens	131578	Reverse	GGGTACCATGGTGTTTCTGG
PDGFRA	Homo sapiens	5156	Forward	CTGGGTTTCCATCCTTGAG
PDGFRA	Homo sapiens	5156	Reverse	TAGTAGGCTTCCTGCGTGG
FAP	Homo sapiens	2191	Forward	TCAAAGAAGTATCCCTTGCTAATTCA
FAP	Homo sapiens	2191	Reverse	GCAATGACCATCCCTTCCTTAC
S100A4	Homo sapiens	6275	Forward	AGGGACAACGAGGTGGACTT
S100A4	Homo sapiens	6275	Reverse	CCCAACCACATCAGAGGAGT

Supplementary table 4

PROTEIN	PRODUCER	CATALOG NUMBER	SPECIES	DILUTION
Phospho-p44/42 MAPK (Erk1/2) (Thr202/Tur204) (20G11)	Cell Signaling	4376	Rabbit	1:1000 in BSA 5% 1h at Room Temperature
Erk1/2 (137F5)	Cell Signaling	4695	Rabbit	1:1000 in milk 5% 1h at Room Temperature
Vinculin	Abcam	ab18058	Mouse	1:1000 in milk 5% 1h at Room Temperature
EGF Receptor (D38B1) XP	Cell Signaling	4267	Rabbit	1:1000 in milk 5% 1h at Room Temperature
pEGFR Tyr1173	Santa Cruz	SC-12351	Goat	1:1000 in BSA 5% O/N at 4oC
HB-EGF	R&D Systems	AF-259 NA	Goat	1:500 in milk 5% O/N at 4oC
E-Cadherin	Cell Signaling	3195	Rabbit	1:250 in BSA 3% O/N at 4oC
Rabbit anti-Goat IgG (H+L) Secondary Antibody, HRP	ThermoFisher	31402	Rabbit	1:2000 in milk 5% 1h at Room Temperature
Goat anti-Mouse IgG (H+L) Secondary Antibody, HRP	ThermoFisher	31430	Goat	1:2000 in milk 5% 1h at Room Temperature
Goat anti-Rabbit IgG (H+L) Secondary Antibody, HRP	ThermoFisher	31460	Goat	1:2000 in milk 5% 1h at Room Temperature
Donkey anti-Rabbit IgG (H+L) Highly Cross-Adsorbed Secondary Antibody Alexa Fluor 555	Invitrogen	A31572	Donkey	1:100 in BSA 3% 45 min at Room Temperature

Supplementary table 5

Adiponectin/Acrp30	IFN-gamma	CCL2/MCP-1
Angiogenin	IGFBP-2	CCL7/MCP-3
Angiopoietin-1	IGFBP-3	M-CSF
Angiopoietin-2	IL-1 alpha/IL-1F1	MIF
Apolipoprotein A1	IL-1 beta/IL-1F2	CXCL9/MIG
BAFF/BLyS/TNFSF13B	IL-1ra/IL-1F3	CCL3/CCL4 MIP-1 alpha/beta
BDNF	IL-2	CCL20/MIP-3 alpha
CD14	IL-3	CCL19/MIP-3 beta
CD30	IL-4	MMP-9
CD31/PECAM-1	IL-5	Myeloperoxidase
CD40 Ligand/TNFSF5	IL-6	Osteopontin (OPN)
Chitinase 3-like	IL-8	PDGF-AA

Complement Component C5/C5a	IL-10	PDGF-AB/BB
Complement Factor D	IL-11	Pentraxin 3/TSF-14
C-Reactive Protein/CRP	IL-12 p70	CXCL4/PF4
Cripto-1	IL-13	RAGE
Cystatin C	IL-15	CCL5/RANTES
Dkk-1	IL-16	RBP4
DPPIV/CD26	IL-17A	Relaxin-2
EGF	IL-18 BPa	Resistin
CXCL5/ENA-78	IL-19	CXCL12/SDF-1 alpha
Endoglin/CD105	IL-22	Serpin E1/PAI-1
EMMPRIN	IL-23	SHBG
Fas Ligand	IL-24	ST2/IL1 R4
FGF basic	IL-27	CCL17/TARC
KGF/FGF-7	IL-31	TFF3
FGF-19	IL-32 alpha/beta/gamma	TfR
Flt-3 Ligand	IL-33	TGF-alpha

G-CSF	IL-34	Thrombospondin-1
GDF-15	CXCL10/IP-10	TIM-1
GM-CSF	CXCL11/I-TAC	TNF-alpha
CXCL1/GRO alpha	Kallikrein 3/PSA	uPAR
Growth Hormone (GH)	Leptin	VCAM-1
HGF	LIF	VEGF
ICAM-1/CD54	Lipocalin-2/NGAL	Vitamin D BP

Supplementary tables title and legend

Supplementary table 1: Summary characteristics of patients included in the study, related to Figures 1, 3, 5. CESC - cervical squamous cell carcinoma; HNSCC - head and neck squamous cell carcinoma; LUSC – lung squamous cell carcinoma; PRAD - prostate adenocarcinoma; PAAD - pancreatic adenocarcinoma; LUAD - lung adenocarcinoma; KIRC - kidney clear cell carcinoma; COAD - colorectal adenocarcinoma; BLCA - bladder urothelial carcinoma; STES - esophagogastric carcinoma; SKCM – melanoma; BRCA - breast cancer.

For UK_HPV positive cohort and for healthy donor blood samples, we do not have access to participant data.

Supplementary table 2: List of STR profiles, related to Figures 1, 2, 3, 4, 5.

Supplementary table 3: List of qPCR primers used, related to Figures 2, 3, 4, 5.

Supplementary table 4: List of antibodies used, related to Figures 3, 4.

Supplementary table 5: List of cytokines tested with Proteome Profiler Human XL Cytokine Array Kit (Catalog #: ARY022B), related to Figure 5.
